# The demographics of developmental hip dysplasia in the Midwestern United States (Indiana)

**DOI:** 10.1007/s11832-015-0636-1

**Published:** 2015-02-19

**Authors:** Randall T. Loder, Cody Shafer

**Affiliations:** 1 Department of Orthopaedic Surgery, Indiana School of Medicine, Indiana University, Indianapolis, IN USA; 2 James Whitcomb Riley Children’s Hospital, ROC 4250, 705 Riley Hospital Drive, Indianapolis, IN 46202 USA

**Keywords:** Developmental hip dysplasia, Demographics, Gender, Race, Laterality, Birth presentation

## Abstract

**Background:**

Today’s society is much more mobile than in the past. This increased mobility has resulted in different marriage/parenting groups. We wished to study the demographics of developmental dysplasia of the hip (DDH) in our area and compare/contrast our findings with those in the literature and specifically look for new findings compared to previous studies.

**Methods:**

A retrospective review of all children with DDH from 2003 through 2012 was performed. The age at first visit, gestational age, pregnancy number, gender, race, and family history of DDH was collected. Statistical significance was a *p*-value < 0.05.

**Results:**

There were 424 children (363 girls, 61 boys). Ethnicity was White in 80.8 %, Hispanic in 13.8 %, Black in 4.0 %, and Indo-Malay and Indo-Mediterranean in 0.7 % each; 66.8 % were unilateral; 14.2 % had a positive family history. The average gestational age was 38.1 weeks; 94.4 % were full term. The child was vertex presentation in 67.6 % and breech in 32.4 %; 52.8 % were delivered vaginally and 47.2 % by Cesarean section. The child was the first-born in 48.3 %. When compared to the birth statistics of our state, there was a higher proportion of Whites and Hispanics with DDH, and a lower, but not inconsequential, proportion of Blacks (*p* = 0.0018).

**Conclusion:**

Mixing of gene pools and infant carrying methods (lack of swaddling or marked abduction) occurring with societal change likely explains the higher than expected proportion of DDH amongst those of Hispanic ethnicity and a lower than expected, but not rare, proportion in those of African ancestry.

**Level of evidence:**

Level IV—retrospective case series.

## Introduction

Developmental dysplasia of the hip (DDH) encompasses a wide spectrum of pathology, ranging from a complete fixed dislocation at birth to asymptomatic acetabular dysplasia in the adult [1–5]. The demographics of DDH have been investigated by many authors [6]. There are no recent studies of the demographics of DDH in the United States, the last ones being from 1968 [7] and 1989 [8]. Today’s society is much more mobile than in the past. This increased mobility has resulted in different marriage/parenting groups, with subsequent mixing of gene pools resulting in gene pool changes. Similarly, with the intermingling of ethnic/racial groups, traditional infant carrying/transport methods may also change. The purpose of this study was to examine the demographics of DDH in our area and compare/contrast our findings with those in the literature and determine if there are any new findings compared to previous studies.

## Materials and methods

All children with DDH treated at the authors’ institution over the 10-year period from 2003 through 2012 were identified by the ICD9 code of 754.3x and appropriate CPT codes (27256, 27257, 27258, 27259, 27146, 27147, 27151, 27156, and 27165). The charts and radiographs were reviewed to confirm the diagnosis. Children with teratologic, neuromuscular, and/or syndromic hip dysplasia were excluded. This study was approved by our local Institutional Review Board.

From the medical records, the age at the first visit to the orthopedic surgeon (which might be slightly older than the age at diagnosis), gestational age, pregnancy number, gender, race, family history of DDH, and treatment modality was collected. Race was classified as White, Black, Amerindian [Native American and Hispanic (Native American/Caucasian mestizos)], Indo-Malay (Asian origins), and Indo-Mediterranean (Middle East and Indian subcontinent) [9]. The demographic data of live births in Indiana from 2003 through 2011 were provided by the Indiana State Department of Health, Epidemiology Resource Center, Data Analysis Team, Indianapolis, Indiana. These were then compared to the proportions of DDH children in this study.

Continuous data are reported as the mean ± 1 standard deviation and categorical data as frequencies or percentages. Differences between groups of continuous data were analyzed using the non-parametric Mann–Whitney (two groups) or Kruskal–Wallis (>2 groups) tests due to the non-normal distribution of the data. Differences between groups of categorical data were analyzed using Fisher’s exact test (2 × 2 analyses) or Pearson’s χ^2^ test (>2 × 2 analyses). Statistical analyses were performed with Systat 10™ software (SPSS, 2000, Chicago, IL, USA). For all analyses, a *p*-value < 0.05 was considered to be statistically significant.

## Results

There were 424 children [363 girls (85.6 %), 61 boys (14.4 %)] with DDH. The racial composition was White in 80.8 %, Amerindian (all Hispanic, no Native Americans) in 13.8 %, Black in 4.0 %, and Indo-Malay and Indo-Mediterranean in 0.7 % each. The DDH was unilateral in 66.8 %; of the unilateral cases, 74.4 % involved the left hip and 25.6 % involved the right hip. The average birth weight was 3,320 ± 574 g (range 624–4,916) and the average gestational age was 38.1 weeks (range 24–42); 94.4 % were full term, 5.1 % pre-term, and 0.5 % post-term. The child was a vertex presentation in 67.6 % and breech in 32.4 %; 52.8 % were delivered vaginally and 47.2 % by Cesarean section. The child was the first-born in 48.3 % and 51.7 % were multigravida births. There was a family history of DDH in 14.2 %. The median age at the first visit to the orthopedic physician was 1.6 months.

There were no differences between those patients with unilateral or bilateral DDH by gender, race, birth weight, gestational age, birth presentation (vertex vs. breech), method of delivery (vaginal vs. Cesarean section), birth order (first-born vs. multigravida), or family history. Similarly, there were no differences within the unilateral group by right versus left hip involvement for all the above same parameters. Finally, there were no differences between those cases with or without a family history of DDH by gender, race, birth weight, gestational age, birth presentation (vertex vs. breech), method of delivery (vaginal vs. Cesarean section), or birth order (first-born vs. multigravida).

Differences were noted by gender for birth weight, presentation, and method of delivery. Girls had a lower birth weight compared to boys (3,300 ± 548 g vs. 3,444 ± 707 g, *p* = 0.025), a higher proportion of vertex presentation (69.7 % vertex, 30.3 % breech) compared to boys (55.2 % vertex, 44.8 % breech) (*p* = 0.034), and fewer Cesarean section deliveries than boys (girls 45.2 %, boys 59.6 %, *p* = 0.046). The only difference by race was for birth weight [White = 3,349 ± 575 g (624–4,961), Hispanic 3,310 ± 451 g (2,466–4,423), Black 2,839 ± 775 g (1,077–3,657), *p* = 0.013].

First-born children had a younger median age at the first visit compared to multigravida births (1.4 vs. 1.9 months, *p* = 0.012). There was a higher proportion of breech compared to vertex presentation (first-born 40.2 % breech, 59.8 % vertex; multigravida 24.3 % breech, 75.7 % vertex; *p* = 0.002) and a higher proportion of delivery by Cesarean section (first-born 46.3 % vaginal, 53.7 % Cesarean; multigravida 58.5 % vaginal, 41.5 % Cesarean; *p* = 0.03).

There were a higher number of Hispanics and a lower number of Blacks with DDH than expected when compared to the proportion of live births in our state (*p* = 1.9 × 10^−6^) (Table 1). Statistical significance remained when excluding the small numbers in the Indo-Mediterranean and Indo-Malay groups (*p* = 3 × 10^−7^).

**Table 1:** Ethnic distribution in children with developmental dysplasia of the hip (DDH)

Race	Number of live births, state of Indiana, 2003–2011^a^		DDH cases	
	*n*	%	*n*	%
White	594,446	77.0	341	80.8
Black	90,054	11.7	17	4.0
Hispanic (Native American/Caucasian admixture)	72,683	9.4	58	13.8
Indo-Malay	4,114	0.5	3	0.7
Indo-Med	10,934	1.4	3	0.7
Total	772,231	100	422	100.0

^a^From the Indiana State Department of Health, Epidemiology Resource Center, Data Analysis Team, 2003 through 2011

## Discussion

Our data are very similar to the other DDH demographic studies regarding gender and laterality. DDH is more common in girls, with 12–30 % bilaterality, and increased in those with breech presentation or delivery [7, 10–46]. Breech presentation or birth in children with DDH ranges from 7.1 to 40 % [6], and was 32.4 % in this series. The percentage of Cesarean deliveries in this study was higher than in the overall state of Indiana from 2003 through 2011 (47.2 vs. 28.9 %, *p* < 0.0001). This likely reflects the higher incidence of breech presentation in children with DDH, as breech babies are much more likely to be born by Cesarean section. DDH is also increased in first-borns [10, 15, 18, 47–50]. In our series, 48.3 % of the children with DDH were first-born compared to the 38.9 % in the general Indiana population (*p* = 0.004). A positive family history is a well-known positive predictor of DDH [11, 12, 15, 17, 26, 34, 51–57]. There was a family history of DDH in 14.2 % of the children in our study.

An interesting new finding in this study was that the number of Black children with DDH was lower than expected, but not inconsequential/rare, when compared to the proportion of live births (4.0 vs. 11.7 %), while the number of Hispanic children with DDH was higher than expected (13.8 vs. 9.4 %). The proportion of DDH in Blacks being ~1/3 of that expected in Indiana is similar to that in New York state [~1/2 of that expected (2.4 vs. 5.1 %)] [7]. DDH was previously considered to be extremely rare in those of African descent [58–66]. Our study suggests that this is no longer true.

This is in contrast to Hispanic children. Hispanics in Indiana are predominantly immigrants from Mexico [67], with a genome admixture of Native American and Caucasian genes [9, 68–70]. The incidence of DDH is high in Native Americans (76.1/1,000) [6]. In a recent review of DDH [6], no study evaluated the incidence/prevalence of DDH in Hispanics. In this study, DDH in Hispanic children was ~33 % greater than expected (13.8 % DDH, 9.4 % normal population), likely due to the presence of Native American genes.

How can these differences be explained? The etiology of DDH is multifactorial, and includes the interaction of both genetic and environmental factors. Genetic mixing in this country of both Blacks and Native Americans with Caucasians can help explain these findings. Blacks in the United States demonstrate a range of 7–20 % admixture with Caucasian genes [71, 72]. The mixing of African genes, which have a lower propensity for DDH when compared to United States Caucasians [58, 64], will raise the prevalence of DDH in American Blacks. The reverse will occur in Hispanics. The mixing of Native American genes with Caucasians, which have a higher prevalence of DDH compared to Caucasians [6], will lower the prevalence of DDH compared to Native Americans, but be higher than that of Caucasians.

An environmental factor involves infant transport and clothing. DDH was very high in Native Americans in earlier studies [6, 73], due to tight swaddling of the infant in most Native American societies. There was a 10-fold increase in DDH (123.0 vs. 12.6) in Canadian Native Americans when infants were placed in a cradleboard [74]. Such tight swaddling is less common in today’s North American societies. Hispanics in this country possess the Native American genes for DDH, but which is no longer exacerbated by tight swaddling. This can explain why the proportion of DDH in Hispanics is higher than in Caucasians, but not as high as in earlier Native American studies. The opposite likely occurs in United States Black children. In traditional African cultures, children are not swaddled, but, rather, abducted across the mother’s pelvis, which protects the infant from DDH. In North America, Black children are not carried in such an abducted position, but, rather, transported in manners similar to Caucasians (e.g., standard baby strollers and car seats), where the hips are not markedly abducted, but neither tightly swaddled.

The influence of environment on genetic factors is well demonstrated by the markedly different incidences of DDH in two different northern circumpolar peoples, the Sámi and Inuit/Eskimos, which have similar environments regarding ambient temperatures. The Sámi, genetically originating from Caucasians [75], traditionally swaddled their young and had a very high incidence of DDH (25–40/1,000) [76–78]. The Inuit/Eskimos have a genetic origin very similar to Native Americans [9, 79, 80]; however, Inuit mothers carry their young in a hood (amauti) inside their parkas, which abducts the hips by straddling the mother’s back. They have an incidence of DDH similar to Caucasians [81]. Finally, DDH is very rare in cultures without swaddling (Southern Chinese, African Bantu, Thailand, North Korea, Sri Lanka [82, 83]).

There are several limitations to this study. First, it does not include all children with DDH in the state of Indiana, as there are likely some children cared for by other orthopedic surgeons outside of our institution. This prevented us from calculating an incidence rate. Next, race/ethnicity was determined from the medical record demographic sheet, which is entered by a clerk, not recorded by a physician. Thus, there is the possibility of some inaccuracy in that variable. Third, the family history of DDH was only that recorded in the medical record as recollected by the parents. Therefore, there is the possibility that some of the positive histories were negative, but also that some of the negative histories were actually positive but not known by the parents and, thus, not recorded. Other variables, such as birth order and birth weight, are likely very accurate.

In summary, this study has confirmed many of the previously known demographic findings for DDH, namely it being more common in girls, left hip, first-born, breech presentation, and Cesarean section delivery. However, it refutes prior studies denoting the extreme rarity of DDH in those of African ancestry. Roper [58] stated that “factors responsible for the primary defects in hip dysplasia are, for practical purposes, absent in the Bantu people”. Griffiths [60] stated that “the apparent rarity of true CDH in Africa remains unexplained”. The percentage of DDH in Blacks in this study (4.0 %) was lower than that of the general population in our state (11.7 %), but, on the other hand, not as extremely rare as quoted by Roper and Griffiths. The percentage of DDH in Hispanics is moderately higher than Caucasians. These two findings likely reflect a convergence of both genetic and environmental factors in the etiology of DDH. A physician evaluating a child for possible DDH of African ethnicity should not assume that the chances of DDH are markedly lower than in other ethnic groups, while the concern for DDH in those of Hispanic ancestry should still be the same, if not heightened.
